# COVID‐digital health literacy and subjective well‐being of students in Ghana: Mediation‐moderation analyses

**DOI:** 10.1002/hsr2.916

**Published:** 2022-11-21

**Authors:** Frank Quansah, Francis Ankomah, Edmond K. Agormedah, Richard S. K. Abieraba, Medina Srem‐Sai, John E. Hagan, Orkan Okan, Kevin Dadaczynski, Thomas Schack

**Affiliations:** ^1^ Department of Educational Foundations University of Education Winneba Ghana; ^2^ Department of Education and Psychology University of Cape Coast PMB Cape Coast Ghana; ^3^ Department of Education SDA College of Education Koforidua Ghana; ^4^ Department of Business & Social Sciences Education University of Cape Coast PMB Cape Coast Ghana; ^5^ Department of Health, Physical Education, Recreation and Sports University of Education Winneba Ghana; ^6^ Department of Health, Physical Education and Recreation University of Cape Coast PMB Cape Coast Ghana; ^7^ Neurocognition and Action‐Biomechanics‐Research Group, Faculty of Psychology and Sports Science Bielefeld University Bielefeld Germany; ^8^ Department of Sports and Health Science Technical University Munich Munich Germany; ^9^ Department of Health Science Fulda University of Applied Sciences Fulda Germany; ^10^ Centre for Applied Health Science Leuphana University Lueneburg Lueneburg Germany

**Keywords:** computer literacy, COVID‐19, health literacy, health status, infodemic, information seeking behaviour, mental health, students

## Abstract

**Background:**

Previous research has established a strong association between COVID‐19 digital health literacy (DHL) and subjective well‐being among several populations, including students. With the growing misinformation and heightened fear of COVID‐19 among persons with an underlying medical condition, several scholars have questioned the direct relationship between DHL and well‐being. This study assessed the moderating roles of information accuracy concerns and the existence of an underlying medical condition among students.

**Methods:**

Using a cross‐sectional design, a multi‐stage sampling approach was used to select 1392 students from senior high schools in Northern Ghana who completed a questionnaire containing information on DHL, information accuracy, subjective well‐being, and underlying health condition, with reported internal consistency coefficients above 0.70. The data which was processed with SPSS version 25, was analyzed using correlation (Pearson and biserial), and Hayes' PROCESS for the moderation and mediation analyses.

**Results:**

A significant positive relationship was found between (a) DHL and subjective well‐being, (b) DHL and information accuracy concerns, and (c) information accuracy concerns and subjective well‐being. However, the prevalence of underlying health condition was negatively associated with information accuracy, DHL, and subjective well‐being. Information accuracy concerns and the existence of an underlying medical condition significantly regulated the relationship between DHL and subjective well‐being.

**Conclusions:**

Demonstrating satisfactory levels of DHL does not necessarily result in improved subjective well‐being. However, emphasis should be placed on whether individuals attach much importance to the accuracy of information retrieved as well as having or not an underlying health condition.

## Patient or Public Contribution

We collaborated with head teachers, teachers and students before and during the administration of the survey instrument across selected public secondary schools in the Northern and Upper Regions of Ghana.

## 1 INTRODUCTION

The outbreak of COVID‐19 pandemic has affected the lives and mental health of students globally.^1^, ^2^, ^3^, ^4^, ^5^, ^6^, ^7^ These psychological consequences are related to the spread of information and misinformation (infodemic) about this epidemic owing to the prevalence of digital social media as the main source of information search among the general public, including adolescents and young adults.^8^, ^9^ Recent studies have found that young adults showed a heightened use of digital media during the COVID‐19 pandemic.^10^, ^11^

Subsequently, digital health literacy (DHL) has become a public health concern.^12^, ^13^ DHL refers to the skills to search, select, understand, appraise online health information and health care and apply the knowledge gained for preventing, addressing, or solving a health problem to improve psychological well‐being.^14^, ^15^, ^16^, ^17^, ^18^, ^19^, ^20^

A substantial literature has indicated that students have reported moderate to high levels of COVID‐DHL (i.e., sufficient DHL) in Germany,^20^ Portugal,^22^ Spain and France,^23^ Slovenia,^24^ and Denmark.^12^ Conversely, a low level of COVID‐DHL has been also reported among students in America^9^, and Europe.^25^ Extant researchers have discovered that higher DHL is associated with subjective well‐being among young adults.^19^, ^26^, ^27^ For example, in China, a positive association between COVID‐DHL and the psychological well‐being of students has been reported.^15^, ^28^ Similarly, Nguyen et al.^16^ in Vietnam established that COVID‐DHL positively influences the subjective well‐being of students. These results suggest that strengthening students' DHL could empower them to actively partake in managing their health by accessing and using accurate health information.

The link between students' COVID‐DHL and subjective well‐being could be explained by the information accuracy screened by the students. However, this relationship has not yet been tested. Information accuracy concern is the extent to which students are concerned about the correctness of the information they search online about coronavirus. Identifying which information is accurate and helpful requires DHL.^26^, ^29^ Accessibility to correct information increases COVID‐19 health literacy.^8^ Nguyen et al.^16^ in Vietnam found a significant positive association between the importance of online information content related to COVID‐19 and the subjective well‐being of students, while Zakar et al.^13^ in Pakistan observed a negative association between students' subjective well‐being and information accuracy. Other researchers have also found the importance of information to be positively related.^13^, ^16^ Based on these results, we assume that information accuracy could mediate the relationship between DHL and the subjective well‐being of students.

Students with “underlying health condition” are at greater risk of COVID‐19 which could affect their subjective well‐being. Accordingly, these students need a level of COVID‐DHL to be able to evaluate COVID‐19 information for use in their daily lives. Also, “underlying health condition” could affect the strength and direction of the relationship between students' COVID‐DHL and subjective well‐being. This relationship has also not yet been tested. However, some studies have established a link between health literacy and health condition.^30^, ^31^, ^32^, ^33^, ^34^ Holt et al.^34^ for example, found that students with an underlying health condition demonstrated lower levels of DHL in Denmark. Liu et al.^30^ in China, on the other hand, found that persons with adequate health literacy are more likely to have reduced chronic disease. Also, in a review, van der Heide et al.^32^ found that many studies reported lower health literacy to be associated with a high occurrence of chronic diseases. Gleaning from the findings of these previous studies, DHL and underlying health condition are related. Likewise, DHL and subjective well‐being. Therefore, there is a likelihood that the underlying health condition would moderate the relationship between COVID‐DHL and subjective well‐being.

In Ghana, digital divide is a major concern among the general public, including young adults and students.^1^, ^35^ The adolescents (including students) in Ghana could have low DHL, which could partly be explained by low socioeconomic and poor health status. Most of the students in Ghana are frequent users of social networks. However, due to the influx of information from varied sources, extracting useful data remain a challenge. Adolescents are not fully equipped to critically analyse information and take appropriate personal and community health decisions.

Overall, there is a paucity of evidence with reference to COVID‐DHL and subjective well‐being among students in Ghana despite a large volume of literature on these phenomena across western countries.^15^, ^16^, ^26^ The study assessed the (a) linear relationship between the pairs of the following variables: DHL, subjective well‐being, information accuracy and prevalence of underlying health conditions, (b) mediating role of information accuracy in the relationship between DHL and subjective well‐being, and (c) moderating effect of the prevalence of underlying health condition in the relationship between DHL and subjective well‐being. The outcome of this inquiry would provide useful information on the vulnerability of students during crises and inform policymakers to provide health educative interventions in schools aimed at strengthening DHL among students to increase their subjective well‐being.

## 2 METHODS

### 2.1 Study participants

This study was cross‐sectional survey, with data gathered from 1392 senior high school (SHS) students from Northern and the two upper regions of Ghana using a multi‐stage sampling approach. Through a random sampling procedure, the Upper West and Savannah regions were first selected. Five schools were then targeted from each region using a cluster sampling technique. This step was followed by purposefully sampling students from each school, considering whether the participants had stayed and/or schooled within the Northern zone of Ghana for more than 10 years. Using G‐power (Version 3.1.9.2) with the actual power of 0.950, seven predictors (including moderation and mediation models), and F‐tests (linear multiple regression: fixed model, *F* ^2^ deviation from zero), a sample size of 1099 was obtained. Furthermore, 30% of the estimated sample size yielded a total sample of 1429. However, 37 responses were not retrieved, leading to a response rate of 97.4%. In the study, 702 (50.4%) and 654 (47.7%) were males and females respectively. Thirty‐six (36) representing 2.6% had diverse sexes. Participants were between the ages of 14 to 25 years, with a mean age of 19 years (*M* = 18.90; *SD* = 1.95).

### 2.2 Study variables

#### 2.2.1 Digital health literacy

The digital health literacy instrument (DHLI) was adapted for this research. The DHLI was originally developed by van der Vaart and Drossaert^19^ but later modified to suit the COVID‐19 situation by the COVID‐HL Network.^36^ In this study, the recommendations from a recent revalidation of DHLI^37^ resulted in a four‐dimensional subscale which includes (a) information searching strategy, (b) adding self‐generated content to online‐based platforms, (c) assessing the reliability of online information, and (d) determining the relevance of online information. Each dimension had three items and was measured on a 4‐point scale (1‐very difficult, 2‐difficult, 3‐easy, and very easy‐4). The recently reported reliability coefficients of the various subscales of the DHLI ranged from 0.758 to 0.834.^37^ Mean scores were computed for each dimension, and these were used for the subsequent analyses.

#### 2.2.2 Subjective well‐being

The subjective well‐being of the participants was measured using the World Health Organization (WHO) well‐being index.^38^ The WHO‐5 index is a unidimensional scale with responses from 0 to 5 (with 0 representing “at no time,” 1 signifying “some of the time,” 2 indicating “less than half of the time,” 3 representing “more than half of the time,” 4 being “most of the time,” and 5 being “all of the time”). The statements comprised: (a) my daily life has been filled with things that interest me, (b) I have felt cheerful and in good spirit, (c) I woke up feeling fresh and rested, (d) I have felt calm and relaxed, (e) I have felt active and vigorous. The participants were required to indicate for each of the 5 statements how they have been feeling over the last 2 weeks at the time of the data collection. The psychometric properties of the WHO‐5 index are sufficient.^39^ The reliability coefficient estimate (Omega ω) reported in this study was 0.815. The sum score was computed for all items and then converted to 100 for use in the follow‐up analyses.

#### 2.2.3 Information accuracy concerns

The information accuracy concerns was conceptualized as the extent to which the participants were concerned about the correctness of the information they search online about coronavirus. The instrument was developed by Gebel et al.^40^ with the following preamble: “This section is about how important various things are to you when you search the internet about coronavirus and related topics. How important is it to you that……”. The instrument contained 6‐items, which are: “the information is verified”, different opinions are represented”, “you quickly learn the most important things”, “the subject is dealt with comprehensively”, “the information is up to date.” The items had responses measured on a 4‐point scale (0‐not at all important, 1‐rather not important, 2‐rather not important, and 3‐very important). A reliability estimate (Omega ω) of 0.771 was reported in this study which was deemed adequate.^41^ The sum score was computed for all items, and this was used in the follow‐up analyses.

#### 2.2.4 Prevalence of underlying health condition

The ‘underlying health condition' variable sought to measure the prevalence of medically diagnosed conditions which makes people vulnerable to coronavirus disease.^42^ The participants in this study were asked to indicate if they had any chronic underlying health conditions. These conditions include lung disease, obesity, cancer, liver disease, diabetes mellitus, neurological disability, mood health disorder, and tuberculosis, among others. If a respondent had any one of the following conditions, a “yes” response was provided whereas a ‘no' response indicated the absence of any chronic health condition.^43^ This variable was treated as categorical.

### 2.3 Procedure

Ethical approval was obtained from the University of Education, Winneba Ethical Review Board with a reference number DAA/P.1/Vol.1/39. In addition, the headmasters of all the senior high schools in the Northern and Upper regions that participated in this particular study gave their approval. Without considering the tribe a student belonged to, the researchers recruited SHS students who had stayed in the Northern and Upper regions for more than 10 years or come from any of the Northern towns. Students who can eloquently speak, read, understand, and write English were used for the study.

After the researchers spoke with 12 research assistants who voluntarily agreed to help in the data collection process, they were taken through the questionnaire from the beginning to the end where each instruction and item were thoroughly explained to them. To facilitate the data collection process, a good rapport was created between the researchers, the research assistants, and members of the school community (headmaster, teachers, and students) through a visit to the selected schools. The researchers took advantage of the visit to explain the rationale of the study to both students and teachers. The researchers then recruited students who were accessible, ready, and willing to be part of the research and were told could opt out any time they willed without any problem. Confidentiality and anonymity were assured, with all COVID‐19 safety protocols observed.

Provision was made for nose masks, hand sanitizers, water, and liquid soap for hand washing, and tissue papers for cleaning wet hands to ensure that students were not exposed to COVID‐19 infection. The questionnaire was given to students to answer during the free periods on the schools' timetable after complying with all ethical issues. It took students 15–20 min to respond to items on the questionnaire. All answered items were taken and kept safely by the researchers.

### 2.4 Statistical analyses

The data for DHL, subjective well‐being and information accuracy concerns were normally distributed. The data were analyzed with different statistical procedures in line with the objectives of the study. First, a correlational analysis (using SPSS version 25) was conducted to establish how the variables (i.e., DHL, subjective well‐being, information accuracy, and prevalence of underlying health conditions) in the study were related. While Pearson‐product moment correlation was used for relationships among DHL, subjective well‐being, and information accuracy, a biserial correlation was used for each of the aforementioned and prevalence of underlying health conditions. Correlation analysis was performed because the focus was to determine bidirectional associations among the variables. Secondly, a simple mediation analysis was performed using the Hayes PROCESS add‐on macro,^44^ to assess the mediating effect of information accuracy in the relationship between DHL and subjective well‐being. Specifically, the Model 4 option was used with multiple predictors using 5000 bootstrap samples. Hayes PROCESS was used for the mediation analysis because it is one of the current ways of testing indirect effect which has shown to be robust in detecting significant indirect effect relative to previous methods such as the Sobel test and Baron and Kenney's approach. Additionally, its use of bootstrap samples helps in estimating unbiased indirect effect with minimal errors. Lastly, simple moderation analysis was also performed with Hayes PROCESS add‐on macro^44^ using the Model 1 option. The moderation analysis used 5000 bootstrap samples and addressed the moderating role of the prevalence of an underlying health condition in the relationship between DHL and subjective well‐being. The bootstrap confidence interval was primarily the basis of interpretation of the mediation and moderation analyses. This approach to moderation analysis was used based on its superiority in testing and probing interaction effects concurrently. The correlation, mediation, and moderation analyses were performed at a 95% confidence level, with all tests being two‐tailed. The correlation analysis was interpreted using a significance level of 0.05 whereas the mediation and moderation analyses were interpreted based on whether the confidence interval had a value of zero.

## 3 RESULTS

### 3.1 Demographic profile of respondents

Table 1 presents the distribution of the demographic characteristics of the respondents, which include sex, age range, class, and whether they had any underlying condition.

**Table 1:** Demographic profile of the students in Ghana

Variables	Levels	Frequency	Percent
Sex	Male	702	50.4
	Female	654	47.0
	Diverse	36	2.6
Age	14–16 years	123	8.8
	17–19 years	840	60.3
	>20 years	429	30.8
Class	SHS 1	322	23.1
	SHS 2	438	31.5
	SHS 3	632	45.4
Do you have any underlying health condition?	Yes	333	23.9
	No	1059	76.1

There were more male than female respondents, with male students constituting 50.4% of the sample and females representing 47%. About 2.6% of the respondents identified themselves as diverse (see Table 1). The largest proportion of the respondents aged between 17 and 19 years (60.3%), and 30.8% were in ages 20 years and above. Very few respondents were between the ages of 14 and 16 years. Third‐year students (SHS 3) constituted a larger percentage of the sample (45.4%), followed by second‐year (31.5%), and first‐year students (23.1%). Although most of the students indicated that they had no underlying chronic condition (76.1%), quite a number of them also reported having an underlying chronic condition.

### 3.2 Relationship among the DHL, subjective well‐being, information accuracy, and prevalence of underlying health conditions

The study explored the relationship existing among the study variables as well as their descriptive statistics as presented in Table 2. The descriptive data showed acceptable values for skewness and kurtosis.

**Table 2:** Correlation matrix, mean, standard deviation, kurtosis, and skewness estimates of the variables

Label	Variables	SIF	SGC	AIR	DRL	DHL	SWB	CIA
SIF	Searching information	1						
SGC	Adding self‐generated content	0.669**	1					
AIR	Assessing information reliability	0.655**	0.684**	1				
DRL	Determining Relevance	0.230**	0.206**	0.144**	1			
DHL	Overall digital health literacy	0.853**	0.858**	0.833**	0.481**	1		
SWB	Subjective well‐being	0.655**	0.412**	0.410**	0.687**	0.583**	1	
CIA	Information accuracy concerns	0.293**	0.308**	0.249**	0.175**	0.538**	0.432**	1
CDI	Existence of chronic disease	−0.047	−0.044	−0.079**	0.035	−0.470**	−0.317**	−0.085**
–	Mean	2.50	2.481	2.553	2.10	2.41	41.02	6.696
–	SD	0.881	0.890	0.876	0.7401	0.650	25.18	4.513
–	Skewness	0.190	0.225	0.098	0.034	0.294	0.429	0.288
–	Kurtosis	−0.806	−0.848	−0.800	−0.884	−0.248	−0.459	0.012

** Correlation significant at *p* < 0.001.

The correlational analysis revealed a significant positive relationship between DHL and subjective well‐being, *r* = 0.583, *p* < 0.001. Similarly, all the dimensions of DHL (i.e., information searching, adding self‐generated content, assessing information reliability, and determining relevance) were positively related to subjective well‐being with coefficients ranging from 0.487 to 0.687 (see Table 2). Information accuracy concerns was also positively related to DHL (*r* = 0.538, *p* < 0.001) and subjective well‐being (*r* = 0.432, *p* < 0.001). It was further found that respondents with underlying chronic health conditions reported lower levels of DHL (*r* = −0.470, *p* < 0.001) and subjective well‐being (*r* = −0.317, *p* < 0.001).

### 3.3 Mediating role of information accuracy concerns in relationship between digital health literacy and subjective well‐being

The study also assessed the mediating role of information accuracy concerns in the relationship between DHL and subjective well‐being. A mediation analysis was performed using subjective well‐being as a criterion variable, information accuracy concerns as a mediator, and the dimensions of DHL as the predictors (i.e., searching for information, adding self‐generated information, assessing information reliability, and determining information relevance). The analysis details are shown in Table 3.

**Table 3:** Mediating effect of information accuracy concerns in the link between DHL and subjective well‐being of students in Ghana

Indicators	Parameters	SIF (X_1_)	SGC (X_2_)	AIR (X_3_)	DRL (X_4_)	DHL
Total Effect	Effect	4.611	0.980	1.754	4.561	10.950
	SE	1.069	1.091	1.087	0.901	0.996
	*t‐*value	4.314	0.899	1.614	5.064	10.993
	BootLLCI	2.514	−1.159	−0.278	2.794	8.996
	BootULCI	6.708	3.119	3.886	6.327	12.904
Direct Effect	Effect	3.162	−0.980	1.515	3.223	5.974
	SE	0.996	1.020	1.008	0.840	0.982
	*t‐*value	3.173	−0.969	1.502	3.836	6.081
	BootLLCI	1.207	−2.989	−0.464	1.575	4.047
	BootULCI	5.116	1.013	3.493	4.871	7.901
Indirect Effect	Effect	1.450	1.968	0.239	1.337	4.976
	BootSE	0.422	0.471	0.450	0.440	0.595
	BootLLCI	0.633	1.112	−0.620	0.501	3.873
	BootULCI	2.297	2.972	1.117	2.238	6.239
Effect Size (Completely standardized indirect effect)	Effect	0.051	0.070	0.008	0.039	0.129
	BootSE	0.015	0.019	0.016	0.013	0.015
	BootLLCI	0.022	0.044	−0.025	0.015	0.101
	BootULCI	0.080	0.117	0.044	0.066	0.159

Abbreviations: Predictors: SIF (X1), Searching for information; SGF (X2), Adding self‐generated content; AIR (X3), Assessing information reliability; DRL (X4), Determining information relevance; DHL, Overall digital health literacy; Criterion Variable (Y), Subjective Wellbeing; Mediator (M), Information Accuracy Concerns; SE, Standard Error; LLCI, Lower Limit Confidence Interval; ULCI, Upper Limit Confidence Interval.

The results, as presented in Table 3, showed that information accuracy concerns significantly mediated the relationship between DHL and subjective well‐being, *β* = 1.337, BootSE = 0.595, BootCI (3.873–6.239). For the specific dimensions of DHL, information accuracy mediated the relationship between three of the domains of DHL (i.e., searching for information, adding self‐generated information, and determining information relevance) and subjective well‐being. Information accuracy, nevertheless, failed to mediate the relationship between assessing information reliability domain and subjective well‐being, *β* = 0.239, BootSE = 0.450, BootCI (−0.620 to 1.117).

### 3.4 Moderating role of underlying health condition in the link between digital health literacy and subjective well‐being

The study additionally sought to establish the moderating role of underlying health conditions in the relationship between DHL and subjective well‐being. Performing a moderation test, DHL served as the predictor, subjective well‐being acted as the criterion variable and the prevalence of underlying health conditions served as the moderator variable. The details of the analysis are presented in Table 4.

**Table 4:** Moderation effect of prevalence of underlying health condition in the link between DHL and subjective well‐being of students in Ghana

	*β*	*SE*	*t*	*p*	BootLLCI	BootULCI	*R* ^2^	*f* ^2^
Constant	28.834	1.630	17.688	0.000	25.636	32.032	0.178	0.216
DHL	8.686	0.911	9.533	0.000	6.899	10.473		
W1	0.815	3.300	0.247	0.805	−5.659	7.290		
Int_1	−4.704	1.887	−2.494	0.013	−8.405	−1.003		

*Note*: Model Summary: *F*(3, 1388) = 39.052, *p* < 0.001.

Abbreviations: W1, Students without underlying health condition; Reference group, Students with underlying health condition Int_1‐ W1*DHL.

As presented in Table 4, the prevalence of underlying health condition significantly moderated the relationship between DHL and subjective well‐being, *β* = −4.704, SE = 1.887, BootCI (−8.405 to −1.003). The moderator contributed about 17.8% of the variability to the relationship between the variables with an effect size of 0.216.

**Figure 1:**
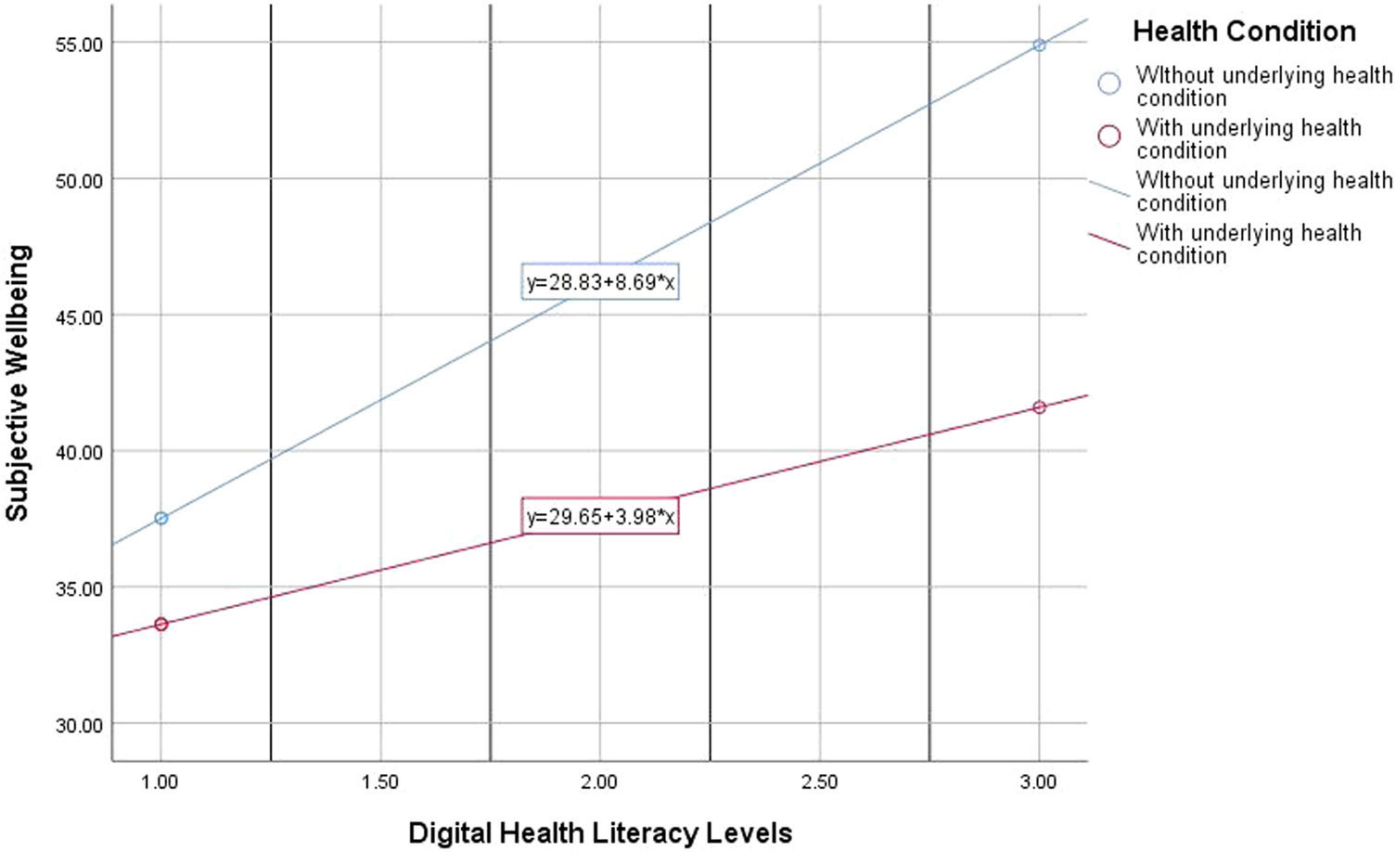
Probing the moderating effect of underlying health condition in the link between DHL and subjective well‐being.

Further probing analysis was conducted by generating and inspecting the moderation curves to understand the nature of the moderation effect (see Figure 1). It was discovered that, for those without underlying health condition, the relationship between DHL and well‐being was steeper (strong) than for those with medical health conditions (weak). In other words, with the same level of DHL, students without health conditions were more likely to experience sufficient levels of well‐being.

## 4 DISCUSSION

One of the objectives of this study was to explore the relationships between DHL, subjective well‐being, information accuracy, and prevalence of an underlying health condition among students in Ghana. It was revealed that the following pairs were positively related: DHL and subjective well‐being; DHL and information accuracy concerns; and information accuracy concerns and subjective well‐being. Summarily, these results imply that higher levels of DHL, subjective well‐being, and information accuracy relate to higher levels among themselves. On the contrary, the prevalence of an underlying health condition was negatively related to information accuracy, DHL, and subjective well‐being. Thus, students with underlying conditions have reduced search for information accuracy, poor DHL, and deteriorated state of subjective well‐being, and vice versa. A plausible explanation could be that their state of health condition has incapacitated their ability to technologically search and evaluate the correctness of COVID‐19‐related information, hence their deteriorated state of subjective well‐being.

Further exploration of whether or not information accuracy could play a surrogate role between DHL and subjective well‐being showed that information accuracy intervenes between DHL and subjective well‐being. Additionally, this result was similar for all the dimensions of DHL except the information reliability domain. This result implies that not only do students have to be digitally literate in terms of obtaining COVID‐19‐related information to enjoy improved subjective well‐being, they equally have to be much concerned about the accuracy of their search content. The findings underscore the importance of students getting accurate information as far as the relationship between DHL and subjective well‐being is concerned. Specifically, information accuracy played a partial intermediary role in the aforementioned relationship, particularly for the determining relevance, and searching information dimensions, as well as the overall DHL. In these situations, information accuracy complemented the influence of DHL on subjective well‐being. The results imply that information accuracy contributed additionally to the effect of DHL on subjective well‐being. Thus, students with high ability and skills in using technological means in searching for health information have a high tendency of improved well‐being. Notwithstanding, when students are more concerned about the precision of their search content, their ability and skills in using technological means in searching enhance their well‐being. This premise suggests the need for students to place much importance on the veracity of COVID‐19 information obtained through the use of digital media. This assertion is pertinent considering the heightened fear associated with the pandemic resulting in its infodemic,^45^, ^46^ and its impact on the well‐being of students.^47^, ^48^, ^49^ Previous studies attributed this infodemic to the influx of digital social media.^8^, ^9^

It is noteworthy that, in the case of self‐generated content as a dimension of DHL, information accuracy completely mediated the relationship. This result signifies that in the absence of information accuracy (i.e., when controlled for), there is no evidence of a direct effect of self‐generated content on subjective well‐being. However, when information accuracy was not controlled for, the relationship between self‐generated content and subjective well‐being was positive. The implication of the results is that, when students have the ability to generate content by themselves, it can only result in better well‐being only if their self‐generated content is accurate. By implication, the call for the training and sensitization of students on the need to ensure that they are consuming accurate information on COVID‐19. Another notable result is the inability of information accuracy to mediate the relationship between the assessment of information reliability and subjective well‐being. Furthermore, controlling for information accuracy, assessment of information reliability had no direct influence on students' subjective well‐being. This counterintuitive result should be further examined by future studies, particularly, during this period of rising interest in DHL.

Overall, information accuracy mediated the relationship between DHL and subjective well‐being. It must be acknowledged that though previous studies have not examined the intermediary role of information accuracy, some have found a link between DHL and information accuracy.^8^, ^26^ Similarly, the link between information accuracy and subjective well‐being has been established.^13^, ^16^ It can be gleaned from these findings a possible mediating role of information accuracy, which supports the findings of this current study. Therefore, it is reasonable to state that DHL, solely, is not a panacea to poor or improved subjective well‐being among students. However, showing concern for the accuracy of health information obtained as well as the exhibition of high ability in the search and evaluation of health‐related information appears to impact subjective well‐being better.

The study further examined the moderating role of underlying health condition in the relationship between DHL and subjective well‐being, which a significant moderation was revealed. Practically, the moderating effect was large. The influence of DHL on subjective well‐being is conditional on a prevailing health condition. For students with the same level of digital literacy, those with an underlying health condition have deteriorated well‐being compared to those without an underlying health condition. This difference in their well‐being became correspondingly very large as their levels of DHL increased. The result implies that with advancement in DHL, the well‐being of students without an underlying health condition improves faster than their counterparts with an underlying health condition. In effect, the underlying health condition is serving as an impediment to the well‐being of students with health conditions, even when they are highly digitally literate in terms of the search and evaluation of health‐related information. Given this, seeking for medical treatment of the underlying health condition is pertinent, considering how detrimental it may be to the subjective well‐being of students. Additionally, other interventions geared towards reducing the effect of the underlying health condition are called for, if improvement in subjective well‐being is so desired. This may also hinder the success of the promotion of health literacy among persons with underlying health conditions. Relating to previous studies^30^, ^32^, ^34^, and Liu et al.^30^ found a relationship between health literacy and health condition. Thus, persons with health conditions have a higher tendency of acquiring more knowledge about their health conditions. With this, such individuals may learn possible ways of preventing or living with their health condition to prevent further health deterioration and hence improved subjective well‐being, contrary to that of the current study where prevailing health conditions rather hindered subjective well‐being. On the contrary, Holt et al.^34^ found that students with an underlying health condition had been associated with lower levels of DHL. This may also imply that the students reported poor DHL possibly because of their precarious health condition which rendered them nonfunctional in their quest for searching and evaluating health‐related information electronically. It is worth noting that in as much as the current finding is compared with previous studies, they are not perfectly comparable. Therefore, further studies are needed for clarity on the identified relationship.

### 4.1 Limitations

The study was limited to students in senior high schools in Northern Ghana. Consequently, the generalization of the findings from the sample to other students' population in Ghana is limited, particularly due to the distinct characteristics of the students in such a geographical location. The study was largely based on self‐reported measures which can affect the validity of the results in situations where participants provide less accurate responses. This notwithstanding, efforts were made by the researchers to ensure that participants provided accurate responses.

## 5 CONCLUSION

The study stresses that demonstrating satisfactory levels of health digital literacy does not necessarily result in improved subjective well‐being. However, much emphasis needs to be placed on whether individuals attach much importance to the accuracy of information retrieved as well as having or not having underlying health conditions. Intervention programs should be directed to students who (a) do not see the need for being concerned about the accuracy of the information they search for and (b) have an underlying medical health condition.

## AUTHOR CONTRIBUTIONS

**Frank Quansah**: Conceptualization; data curation; formal analysis; investigation; methodology; project administration; software; validation; visualization; writing – original draft; writing – review & editing. **Francis Ankomah**: Investigation; methodology; validation; visualization; writing – original draft; writing – review & editing. **Edmond K. Agormedah**: Investigation; methodology; validation; visualization; writing – original draft; writing – review & editing. **Richard S. K. Abieraba**: Investigation; methodology; validation; visualization; writing – original draft; writing – review & editing. **Medina Srem‐Sai**: Investigation; methodology; validation; visualization; writing – original draft; writing – review & editing. **John E. Hagan**: Conceptualization; funding acquisition; investigation; methodology; project administration; resources; supervision; validation; visualization; writing – original draft; writing – review & editing. **Orkan Okan**: Investigation; methodology; validation; visualization; writing – original draft; writing – review & editing. **Kevin Dadaczynski**: Investigation; methodology; validation; visualization; writing – original draft; writing – review & editing. **Thomas Schack**: Funding acquisition; investigation; methodology; resources; validation; visualization; writing – original draft; writing – review & editing.

## CONFLICTS OF INTEREST

The authors declare no conflicts of interest.

## INSTITUTIONAL REVIEW BOARD STATEMENT

The study was approved by the Institutional Review Board of the the University of Education, Winneba, Ghana with document number DAA/P.1/Vol.1/39.

## INFORMED CONSENT STATEMENT

Written informed consent was taken from all study participants before data collection.

## TRANSPARENCY STATEMENT

The lead author John Elvis Hagan affirms that this manuscript is an honest, accurate, and transparent account of the study being reported; that no important aspects of the study have been omitted; and that any discrepancies from the study as planned (and, if relevant, registered) have been explained.

## Data Availability

Anonymized data is available upon reasonable request through the corresponding author and takes complete responsibility for the integrity of the data and the accuracy of the data analysis.
